# Can Dogs’ Origins and Interactions with Humans Affect Their Accomplishments? A Study on the Responses of Shelter and Companion Dogs during Vocal Cue Training

**DOI:** 10.3390/ani11051360

**Published:** 2021-05-11

**Authors:** Maria Luiza A. Fonseca, Angélica S. Vasconcellos

**Affiliations:** Department of Biological Sciences, Pontifical Catholic University of Minas Gerais, Belo Horizonte 30535-901, Brazil; marialunesfonseca@gmail.com

**Keywords:** dog cognition, dog–human interactions, life history, shelter dog, training, welfare

## Abstract

**Simple Summary:**

The life history of animals is an important aspect to be considered when behavior, welfare, or cognition is investigated. Here, we aimed to study the behavioral responses of dogs with different origins—shelter and companion dogs—when learning two basic vocal cues (“sit” and “paw”), as well as possible associations between dogs’ responses and the behaviors of the trainers. Shelter dogs responded to more cues per session, were faster in their responses, and needed fewer repetitions of cues to respond than companion dogs. Moreover, shelter dogs spent more time wagging their tails during the sessions. Some behaviors of the trainers were associated with dogs’ behaviors—the use of a reproachful tone of voice, although associated with dog performance, was also linked to the exhibition of behaviors indicative of discomfort on the part of dogs. On the other hand, the use of a neutral tone of voice and laughter, in addition to being connected to performance, was also associated with behaviors indicative of excitement. These results suggest that shelter dogs’ capacities for learning vocal cues are not affected by the shelter environment. Furthermore, shelter dogs showed greater interest in the sessions than companion dogs, possibly due to the social deprivation in their routine. Our outcomes also point to a connection between friendly interactions during training and the dogs’ performance, and possibly their emotional state. The quality of the interactions seems to affect dogs’ performance and welfare more strongly than their origins.

**Abstract:**

The inclusion of life history as a possible influential factor is pivotal in studies on behavior, welfare, and cognition. Shelter dogs have usually experienced a life involving poor social interactions with humans. Thus, we aimed to investigate the behavioral responses of shelter dogs (SDs) and companion dogs (CDs) during the training of two vocal cues (“sit”, “paw”), as well as the possible associations between their responses and the behaviors of trainers. We studied 15 SDs and 15 CDs in up to eight five-minute training sessions. Dogs’ and trainers’ behaviors were recorded and analyzed (through GLM, GLMM, correlation and Mann–Whitney tests). Shelter dogs responded to more cues per session, with shorter latencies and fewer repetitions of cues. Moreover, SDs spent more time wagging their tails. Dogs’ sex and trainers’ behaviors were also associated with differences in dogs’ responses. The use of a reproachful tone of voice was associated with a greater number of cues responded to, shorter latencies, and fewer repetitions of cues. However, this type voice/discourse was also linked to a greater exhibition of non-training behaviors (e.g., exploring the room or jumping on the trainer), and to dogs spending less time next to the trainer and wagging their tails. On the other hand, the use of a neutral tone of voice and laughter, besides being linked to performance, was also associated with longer durations of tail wagging. Furthermore, the duration of the trainers’ orientation to dogs was correlated with the orientation of the dogs to the trainers. Our data suggest that, even when having experienced social deprivation from humans, SDs’ capacities to learn vocal cues were preserved, possibly due to ontogenic homeostasis processes. Shelter dogs’ greater interest in the sessions may be also credited to their socially-deprived routine. Our outcomes also point to an association between friendly interactions during training and dog performance and excitement, which suggests that such interactions may have the potential to improve SD welfare.

## 1. Introduction

Cognition is a system involved in knowledge acquisition, and people who own dogs tend to claim that these animals have great cognitive skills [1]. Dogs are widely used in cognition studies, mainly due to their cognitive capacities, which, depending on the task, may be greater than those of other species, such as wolves and chimpanzees [2,3]. Throughout domestication, dogs went through a process of selection of skills for interacting and communicating with humans, including relying on human gestures and making eye contact [4,5], which may have contributed to the development of their cognitive resources. For instance, dogs follow human pointing gestures, an ability that has been related to the absolute brain size of the species [6,7]. In tasks which consisted of understanding social cues given by humans to find hidden food, although dogs tended to act strongly based on olfactory cues, their performance was evident [8,9].

Dogs evolved while coexisting with humans, and friendly interactions between the two species are known to promote positive effects on canine welfare [10,11]. The selection for canine characteristics to facilitate the interactions of these animals with humans, as well as the intense coexistence between the two species may have been responsible for stimulating the development of attachment bonds in dogs. Dogs tend to play and explore an unfamiliar environment more when their owners are present [12]; when dogs feel threatened by a stranger, both their heart rate and its variation increase if the owner is not present, whereas his/her presence reduces these increases [13]. Taking effects like these into consideration, researchers state that attachment bonds between dogs and humans are similar to those developed between human babies and their mothers [14]. From this perspective, the separation of dogs from their attachment figures—their owners—may favor the development of separation-related problems, which in turn, increases the chance of developing a pessimistic cognitive bias, an effect common in pet dogs, and also reported in dogs from reallocation centers [15].

The life history of dogs was shown to affect relevant aspects of their social competence, such as dog’s persistence on gazing behavior, or at being close to humans [16]. For instance, shelter and companion dogs looked more at human faces, and spent more time close to humans, compared to dogs that lived on the streets [16]. Shelter dogs performed well in tests that involved vocal cues but had difficulty learning visual cues [17]. In another study, using an A-not-B task, in which dogs had to learn that a reward was relocated and reach it [18]—shelter dogs did not differ from companion dogs in the number of correct responses in the training stage, but learned more slowly and had a greater number of incorrect choices during the test stage. Such differences might be related to the fewer opportunities shelter dogs have during their life to observe people’s behaviors, or to be rewarded for following their directions. Companion dogs live with humans; therefore, they are constantly being stimulated by humans, and some of their responses in the presence of certain stimuli increase because these have led to a high probability of reinforcement in the past [19]. On the other hand, shelter dogs do not underperform companion dogs in all abilities. No differences were found between these dogs in discriminating generous human attitudes from selfish ones [20], an important skill for social interaction. Shelter dogs have also shown contagion with human yawning, although this phenomenon depends on context and the dog’s excitement [21].

Friendly human–animal interactions, understood as mutual interactions between human beings and animals that have a relaxing effect on animals, have been recommended in order to improve the welfare of animals [22]. A type of interspecific interaction that is common between people and dogs is training [23]. Training is usually performed with the use of operant conditioning techniques, which consists in an individual associating his/her actions (e.g., giving a paw) with a consequence (e.g., receiving a reward), and this association usually has effects on the probability of this individual repeating that action [24].

A previous study by our team [10] found out that positive reinforcement training, a type of operant conditioning, had relaxing effects on dogs and wolves, an effect which varied with trainer. Studies have pointed to human influences on dogs’ learning processes. Although some studies have found no effect of training method on the behavior or welfare of animals [25], some have raised evidence that the use of punitive methods in training may, for instance, affect a dog’s ability to learn a new task [26], or increase their stress levels (e.g., [27,28]). A dog’s ability to attend to a cue may also be influenced by the vocal information the trainer includes before the cue (e.g., saying the dog’s name or an unknown word [29]); even if the dog is familiar with the cue, this vocal information may reduce the dog’s performance. Studies have been conducted on the motivation and effects of people’s tones of voice when communicating with dogs (e.g., [30,31,32]) but as far as we know, no studies have investigated associations between trainer vocal behavior and the behavioral responses of dogs during training. A study of vocal cue learning, considering the effect of dog origin and possible associations between trainers’ behaviors and dogs’ responses, has the potential to improve our understanding of dog learning processes and abilities.

The purpose of this study was to investigate how: (a) the origin of dogs (in terms of their interactions with humans), and (b) trainer behavior correlate with dogs’ abilities to learn vocal cues. We hypothesized that shelter dogs would perform differently from companion dogs in the tasks. We also expected some behaviors of trainers to be correlated with animals’ responses. More specifically, our predictions for this study were: (a) shelter dogs would need a greater number of sessions to learn the cues, more repetitions of cues, and would show greater latencies in responding to them; (b) both shelter and companion dogs would perform better in sessions when the trainers exhibit friendly behaviors for longer. Shelter dogs responded to more cues per session, with shorter latencies and fewer cue repetitions. Furthermore, these dogs showed more excitement during the sessions, maybe due to their history of social deprivation. However, the data supported our second prediction—the use of a neutral tone of voice and laughter was associated with dogs’ performance and excitement. Our outcomes suggest that friendly interactions with humans may contribute to dogs’ performance and—possibly—welfare.

## 2. Materials and Methods

This study was approved by the Ethics Committee on the Use of Animals of the Pontifical Catholic University of Minas Gerais, Belo Horizonte, Brazil (Protocol 15/2019). The owners of all dogs allowed their animals to participate in the study by signing a Free and Informed Consent Form.

### 2.1. Subjects

Fifteen shelter dogs (SDs) and 15 companion dogs (CDs) were studied (Table 1). The average age of the sample was 4.34 years (±0.47 SE; 4.93 ± 0.85 SE for CDs and 3.71 ± 0.33 SE for SDs). Shelter dogs’ mean weight was 11.6 kg (±0.70 SE); in the CD group, the mean weight was 6.7 kg (±0.78 SE). The training sessions of one SD were considered non-standard, since cues were asked at intervals shorter than five seconds (please see procedures); therefore, this dog was excluded from the analyses (female, 4 years old, mixed breed).

The SDs studied lived in three shelters for abandoned/stray dogs in the city of Belo Horizonte—Veterinária Ministério Arca de Noé, Pousada Cão Alegria, and Casa de Passagem Neverland, and had a routine with a low frequency of interactions with humans. Our inclusion criteria for SDs were that the dogs should not know the proposed cues, nor be used to the physical and/or cognitive activities typical of training, and should have been living in the shelter for at least six months. The CDs also lived in the city of Belo Horizonte and were recruited through personal contacts. These dogs had a higher level of interaction with humans compared to SDs, as they were living as pet dogs. The inclusion criteria for these dogs were that they should not know the proposed cues, nor be used to the physical and/or cognitive activities typical of training, and should have been living with their owners for at least six months. All dog-owners/shelter-keepers were instructed not to practice the cues with the dogs during the period in which the sessions were being held.

### 2.2. Procedures

All dogs were submitted to training sessions to learn to sit and give their paw in response to a cue (cues “sit” and “paw”) from October 2019 to February 2020. All training sessions of SDs took place in the shelters in which they were housed, so that they did not have to travel to take part in the sessions. These dogs were trained in a quiet room, in the presence of only the trainer responsible for conducting the session. The CDs were trained in their respective homes, in similar conditions. Two trainers were responsible for conducting the training sessions; both trained dogs from the two groups (SDs and CDs), but each dog was trained by just one trainer. Trainer 1 (T1) conducted 10 SD and 10 CD training sessions, whereas Trainer 2 (T2) conducted training of four SDs and five CDs.

All 5-min training sessions were recorded for later analysis. The sessions were conducted using operant conditioning in a naturalistic context. A clicker was used as a secondary reinforcer, being activated as soon as the dog responded correctly to the cue, prior to the offering of the reward (a meat snack). Before starting the first session with each dog—in order to promote the association of the clicker sound with the reward—the clicker was activated 10 times, followed each time by the offering of the snack to the dog.

Prior to the beginning of each session, all dogs went through a three-minute habituation stage in the training room (after their owners’ left in the case of CDs, or their keepers’ in the case of SDs). After this period, a 1-m^2^ satin vinyl foam sheet was spread on the floor to serve as a reference station, and the trainer called the dog. If the dog did not approach (i.e., if the dog was not motivated enough to interact) the session would not occur. However, this did not happen in any of the sessions. The dogs, when called, approached right away, mostly wagging their tails. After the dog was on the station, the training session began. The cues “sit” and “paw” were then continuously asked, following a previously-randomized order. For the “sit” cue, the trainer stood in front of the dog and pronounced the cue, maintaining eye contact with him/her; if the dog voluntarily sat down, the trainer captured the behavior with the clicker and offered the reward (i.e., free shaping [33]). If the dog did not sit after five seconds, the cue was repeated. If that was still not effective, modeling would take place—the trainer would gently press the dog’s hip to the floor, in order to stimulate him/her to sit down. To teach the “paw” cue, the trainer remained kneeling facing the dog, held the snack in her closed hand and showed the hand to the dog, so that the dog would try to reach the snack with his/her paw [34,35]. The same five-second rule was followed. If this strategy was not effective, the trainer repeated the cue while holding the dog’s paw. The following cue could only be requested after a 5-s interval. Vocal cues were stated clearly and calmly, and each correct action by each dog was marked by the sound of the clicker, followed by the offering of the snack. At the end of the session, the clicker was triggered for the last reward of the day, followed by snacks, praise, and pats. No dog had more than one training session on the same day.

The learning criterion adopted was two consecutive sessions with at least 60% correct responses. This learning criterion was chosen based on previous studies in which SDs showed poor performance, when compared to CDs [17,18,19]—a more demanding criterion could have prevented us from drawing consistent conclusions from our data. When the dog reached this criterion, we considered that he/she had learned the cues, and his/her training sessions were ended. For logistical reasons, a maximum of eight sessions were run per dog. Thus, if a dog was not able to respond correctly to 60% of the cues by the end of the eighth session, the training was ended nevertheless.

### 2.3. Data Analysis

All videos recorded during the sessions were coded (Solomon Coder beta version 19.08.02; 2019, András Péter) for dog behavior through focal sampling and continuous recording by the same observer (MLAF), who was familiar with the animals and the coded behaviors. In addition to coding the dogs’ responses to the cues, the distance between dog and trainer, dog orientation (the orientation of its head deviating less than 10° from trainer’s face; [10]), the time the dogs spent wagging their tails (as indicators of dogs’ excitement/interest in the interactions [36,37]), and non-training behaviors (NTBs) exhibited by the animals were also recorded. Non-training behaviors were all behaviors that did not contribute to the training process and took the animal, or its attention, away from the trainer. These behaviors were considered to be indicators of excessive excitement, lack of attention/dispersion, boredom, or of an increase in stress levels, indicating that the animal was not focused on the activity [10]. The observed NTBs were (1) jumping on the trainer: the animal is either standing on his/her hind legs or jumping with his/her four legs leaving the ground, and touching the trainer with his/her forelegs; (2) leaving: the animal moves away from the trainer, abandoning the interaction, and (3) exploring: the animal sniffs the ground or the walls. To confirm scoring consistency, 20% of videos were recoded, and the records were correlated [38,39]; the mean Spearman’s rank correlations (rS) per video were 0.83.

As a step further, in order to investigate possible correlations between trainers’ behaviors and dogs’ responses, the behaviors of trainers were also coded, through continuous recording and behavioral sampling of: face orientation, dog petting, and vocal behaviors (laughter and types of speech used–reproachful, gentle, neutral; Table 2). The acoustic parameters of these three types of speech have been proven to be distinct from each other (low frequency; high frequency; peak frequency; average power; delta time and number of speeches; [40]). Both trainers used all types of speech.

Dog behavioral data were analyzed through generalized linear models (GLMs) and generalized linear mixed models (GLMMs), with the Poisson distribution. Minimal adequate models were obtained using the iterative method. We used “lme4” [41], “MASS” [42], and “car” [43] packages to fit GLM/GLMM models in R statistical software, version 3.5.2 [44]. All results were analyzed based on statistical significance (α ≤ 0.05).

We structured our analysis into separate models: GLM1 (N = 29), to investigate the effects of dog origin and sex, as well as trainer identity, on animals’ performance; GLMM2, to evaluate the effects of dog origin and sex on their general behavioral responses; GLMM3, to investigate the associations between trainers’ behaviors and dogs’ performance; and GLMM4, to check for possible associations between trainers’ behavior and dogs’ general behavioral responses. The parameters we considered to be indicators of dog performance were: (a) mean latency to respond to the cues at the first request; (b) mean number of repetitions per cue; (c) number of sessions necessary to reach the learning criterion and (d) to learn each cue; (e) number of cues responded to; (f) number of dogs that reached the learning criteria. As general behavioral responses (non-performance parameters), we considered (a) the mean duration of tail wagging; (b) the time the animal spent within 1 m of the trainer; (c) the duration of non-training behaviors; and (d) the time the animal spent oriented towards the trainer. All GLMM models were carried out considering all dogs’ sessions (repeated measures). The fixed effects and response variables of each model are described in Table 3.

Threee of the fixed effects—the time the trainer spent oriented to the dog, the time the trainer spent using neutral, and gentle speech—did not fit in all GLMM models. Therefore, we used Spearman Correlation test to check for correlations between these explanatory variables and the response variables—the mean duration of tail wagging; the time spent within 1 m of the trainer; duration of NTB; time the animal spent oriented towards the trainer, mean number of cue repetitions; mean latency to respond to the cues; mean number of cues responded to per session.

Considering that the average size of the SDs was different from that of the CDs, and that dog size has the potential to influence dog behavior [45], in order to control for a possible influence of this variable on the behaviors of the study dogs, we ran a GLM model to investigate the effects of dog weight on their performance.

Finally, we compared (using the Mann–Whitney Test) the sessions conducted by the two trainers in terms of the duration of each type of speech (reproachful, gentle, neutral), and laughter.

## 3. Results

### 3.1. Dog Performance

**Figure 1:**
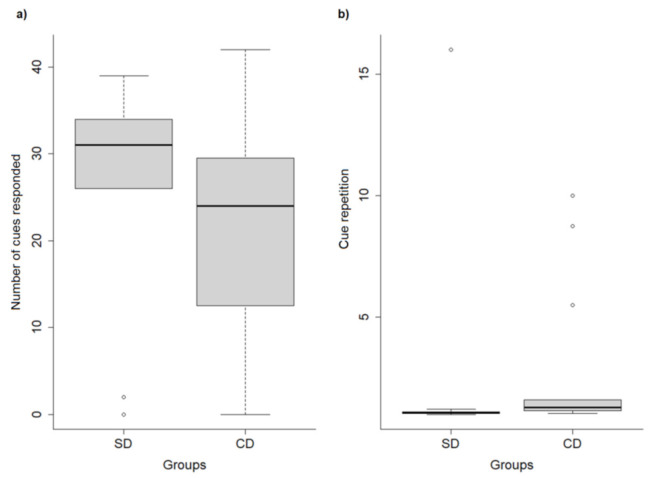
Number of cues responded to (**a**), and cue repetition (“sit” and “paw”) (**b**) in the last training sessions of shelter dogs (SDs) and companion dogs (CDs). Bold horizontal lines represent medians; gray boxes represent quartiles; and thin horizontal lines depict minimum and maximum values.

**Figure 2:**
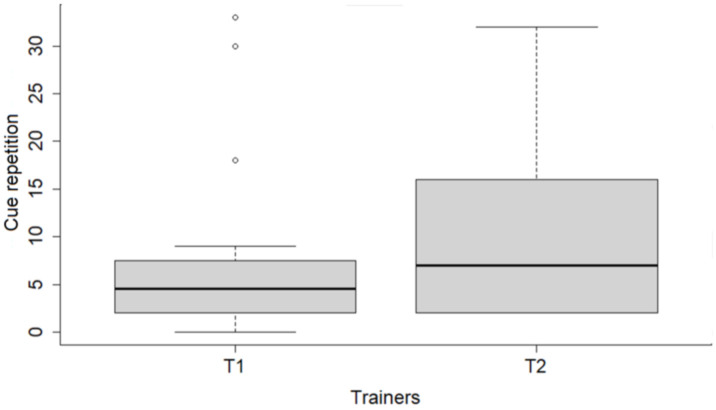
Cue-repetitions in the last training sessions of shelter and companion dogs with Trainer 1 (T1) and Trainer 2 (T2). Bold horizontal lines show medians; gray boxes represent quartiles; thin horizontal lines show minimum and maximum values.

Dog weight had no effect on their performance (all *p*-values > 0.05; Table S1—Supplementary material). The final reduced models for dog performance (GLM1) are shown in Table 4. Shelter dogs responded to more cues (Figure 1a), needed fewer repetitions of cues to respond (Figure 1b), and responded with shorter latencies, compared to CDs. No other variable related to performance pointed to an effect of dog origin. Male dogs presented longer latencies, needed more cue repetitions to respond, and responded to fewer cues than females. Trainer identity also affected dog performance in the last session—Trainer 2 obtained dog responses faster, but had to repeat cues more often than Trainer 1 (Figure 2), and had fewer cues responded to.

### 3.2. Dogs’ General Behavioral Responses

**Figure 3:**
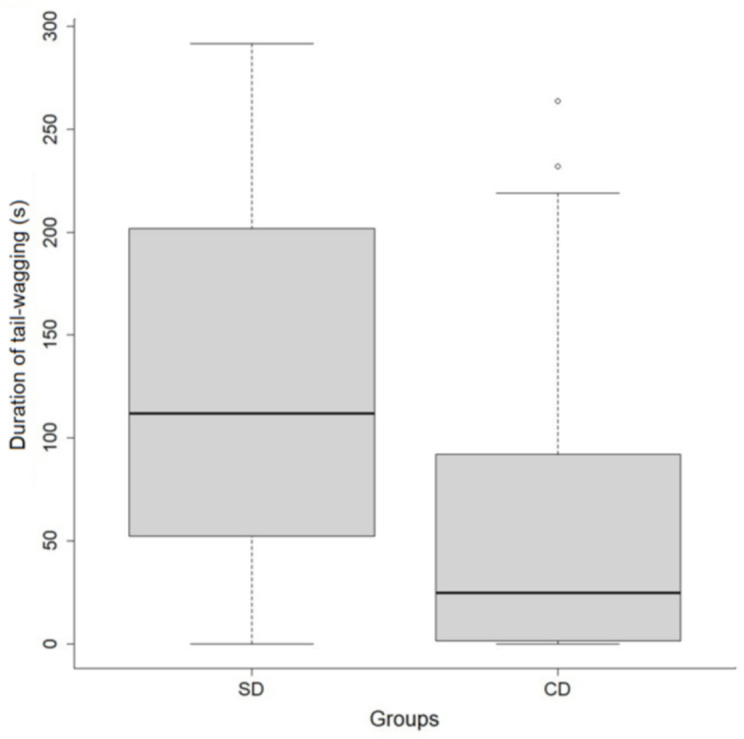
Duration of tail-wagging for shelter dogs (SDs) and companion dogs (CDs) during training sessions. Bold horizontal lines represent medians; gray boxes represent quartiles; thin horizontal lines represent minimum and maximum values.

The final reduced model for the effects of dogs’ origin and sex on their general behavioral responses (GLMM2) is shown in Table 5. Shelter dogs spent more time wagging their tails during the sessions than CDs (Figure 3). No other recorded behavior showed an effect of dogs’ characteristics on the response variables.

### 3.3. Associations between Trainers’ and Dogs’ Behaviors

**Figure 4:**
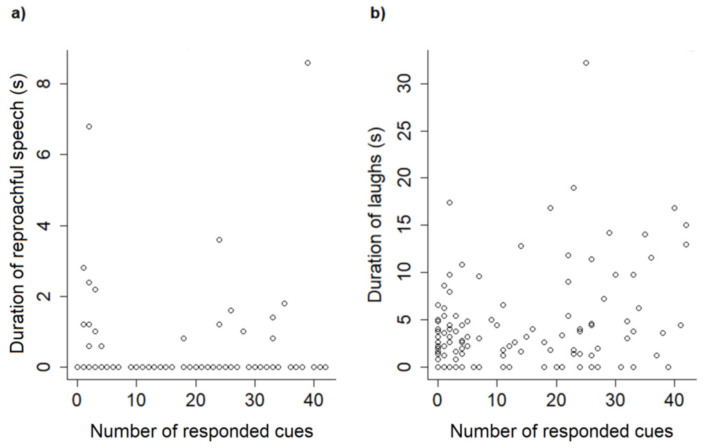
Dispersion of the duration of trainers’ reproachful speech (**a**) and laughter (**b**) during training sessions with shelter and companion dogs as a function of the number of cues responded to per session.

The final reduced models for the investigation of associations between trainers’ behaviors and dogs’ performance (GLMM3) and general behavioral responses (GLMM4) are shown in Table 6 and Table 7. The use of reproachful speech by trainers was associated with shorter latencies in the responses to the cues, fewer repetitions of cues, and more cues responded to per session (Figure 4a). On the other hand, the time trainers spent laughing during the sessions, although positively correlated to latencies, was also linked to fewer repetitions of cues and a greater number of cues responded to per session (Figure 4b). The time trainers spent petting the dogs during the sessions was associated with fewer cues responded to; however, the size of this effect was negligible (estimate −0.01).

**Figure 5:**
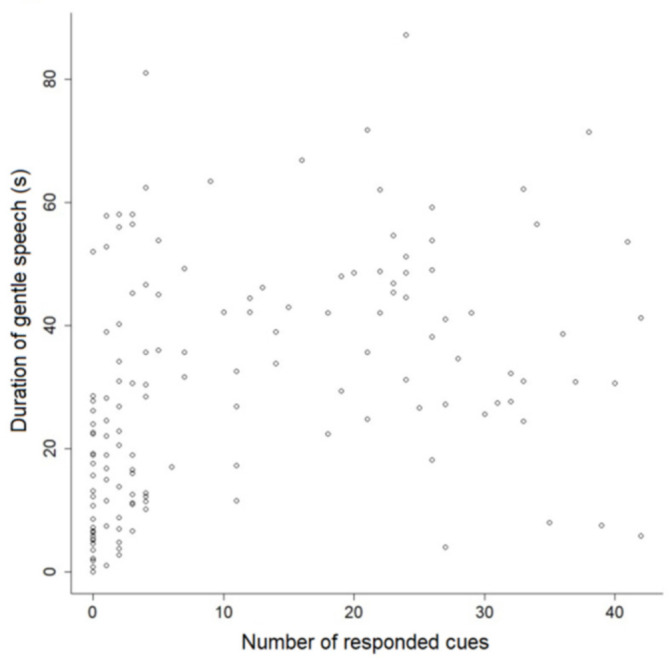
Dispersion of the number of cues responded to by shelter and companion dogs per training session as a function of the duration of trainers’ gentle speech.

The duration of the trainers’ orientation to dogs correlated negatively with the number of cues responded to per session (*p* < 0.001, rho = −0.32) and positively with dogs’ latencies in responding (*p* = 0.03, rho = 0.15). On the other hand, there was a positive correlation between the duration of gentle speech and the number of cues responded to per session (*p* < 0.001, rho = 0.49; Figure 5).

The use of reproachful speech by trainers was linked to shorter durations of tail wagging, less time in which dogs were within 1 m of the trainer, and more time dogs spent exhibiting NTB. On the other hand, the use of neutral speech was positively associated with tail wagging (Table 6). The duration of the trainers’ neutral speech correlated negatively with the time the dog spent oriented to the trainer (*p* = 0.03, rho = −0.17), but trainers’ orientation to dogs correlated positively with the time in which dogs were oriented to trainers (*p* = 0.02, rho = 0.19). Trainer 2 used reproachful speech for longer than Trainer 1 (w = 2074, *p* = 0.02), while Trainer 1 used gentle speech for longer (w = 2945.50, *p* = 0.01), as well as laughing more (w = 3908.50, *p* < 0.01).

## 4. Discussion

In this study, we aimed to assess the responses of shelter and companion dogs during operant conditioning training to learn two vocal cues, considering the associations between trainer behavior and dogs’ responses. We found dog origin (shelter vs. companion), dog sex, and trainer (Trainer 1 vs. Trainer 2) to be associated with differences in dog performance. Shelter dogs responded to more cues, with shorter latencies, and fewer repetitions of cues required for them to respond than CDs. Male dogs took more time, needed more cue repetitions to respond, and responded to fewer cues than females. We also found correlations between the behavior of the trainers and dogs’ performance and general behaviors. Although the use of a reproachful speech was positively associated with training performance (lower latencies, fewer cue repetitions, and more cues responded to), it was also linked to shorter durations of dogs within one meter of the trainer and wagging their tails, and to longer durations of NTB. On the other hand, the use of neutral and gentle speech was associated with a greater number of cues responded to, and more time spent tail-wagging. Moreover, the orientation of trainers to dogs was positively associated with the orientation of the dogs to the trainers.

Some studies have found SDs to perform similarly to CDs (e.g., [18,46]). The SDs in our study, however, performed better than CDs in three out of five of the parameters measured: the number of cues responded to, latency in responding to cues, and the number of cues repeated. This indicates that, despite their routine with a low rate of interaction with humans, the ability of shelter dogs to learn basic vocal tasks was not impaired. One possible explanation has to do with ontogenic homeostasis. Ontogenic homeostasis is a process in which, despite living in a poor environment or having defective genes, an individual somehow develops normally [47]. Such phenomenon applies usually to systems or abilities that are essential for survival, such as in social cognition. An example of such an effect is the classical study by the Harlows involving the deprivation of contact with the mother in infant rhesus monkeys, who, although exhibiting abnormal social and sexual behaviors, presented normal physical development [48]. Another study that reported this effect—this one on human cognitive development—found that, despite the nutritional deprivation of mothers during their pregnancy throughout the Dutch famine of 1944–1945, their children did not show differences in mental performance in relation to children whose mothers did not experience deprivation, even nineteen years later [49]. This buffering effect on cognition development/maintenance might be responsible for the good performance of SDs in our study. However, as ontogenic homeostasis processes refer to the deprivation animals suffer while developing, and we did not have information about the age at which the study shelter dogs arrived at the shelters, this explanation may not apply to all of them. Nevertheless, the deprivation of human contact in the experience of SDs may have predisposed them to interact with unfamiliar people (i.e., the trainers) more than CDs did [11,12,50,51]. Another factor that might have contributed to the difference in performance between SDs and CDs in our study is the separation of CDs from their owners during training. Although the protocol was standardized for both dog groups, CDs might have been affected more by the procedure due to a strong attachment to their owners [12,14]. However, we think this is improbable, since their reactions when the owners left consisted mostly of looking at the door for a few seconds; none of the CDs remained by the door or refused to take part in the training sessions.

In addition to the differences in performance discussed above, the duration of tail wagging—usually reported as an expression of a positive emotional disposition [52,53,54]—was greater for shelter dogs and had no relation to the identity of the trainer. The novelty—and possibly the value—of training interactions with humans was possibly greater for SDs than for CDs, regardless of with whom the interaction took place. This outcome is in line with a study [20], in which SDs, during the extinction phase of previously-taught behaviors, spent more time next to the experimenter than CDs did, possibly because they were more willing to interact with humans, or because the human presence somehow buffered the frustrating effect of not being rewarded for a behavior [51,55]. In another study, when approached by an unfamiliar experimenter, although SDs displayed more fear-related behaviors towards the experimenter, they stayed close to her for longer periods compared to CDs [56]. Such results suggest a greater need among SDs for contact with humans [57].

We found differences in some parameters related to performance between sexes: although females responded to more cues, at a faster speed, and with fewer repetitions, males wagged their tails for longer during the sessions. Males and females have been reported to excel in different tasks—males have shown greater performance in maintaining eye contact with experimenters and in short-term memory tests [58], whereas females were better at leaving a maze [59]. The inferior performance of males in our study may be related to their possibly lower trainability, as already reported [60]. One could also suggest that, as males present more separation-related problems [61], their separation from the owners during the sessions might have affected them more than it affected females. However, their longer duration of tail wagging suggests that this was not the case. An alternative explanation for this difference could be that males responded to a smaller number of cues because they were less attentive or focused than females, possibly because they were more excited.

Dog performance was associated with the type of speech used by the trainers during the sessions, as well as their laughing. The use of reproachful speech was connected to performance—shorter latencies and fewer cue repetitions, and a greater number of cues responded to. However, this type of discourse was also correlated with dogs’ general behaviors, especially those indicative of internal states. In sessions when this type of speech was used for longer, dogs wagged their tails less, stayed within 1 m of the trainer for less time, and exhibited more NTB (behaviors that suggested the animals were not focused, or not interested in the interactions). Although most training procedures were based on positive reinforcement (i.e., when the consequence of an individuals’ actions is being rewarded, which has proven to be beneficial for reducing stress [10]), it is possible that dogs perceived reproachful speech as a punishment [23] and, although responding to the cues, were emotionally affected by the interaction, as some studies have shown (for reviews, see [25,62]). On the other hand, the use of gentle speech was related to a greater number of cues attended to; neutral speech was positively associated with tail wagging, and laughter—although linked to increased latency—was also associated with fewer cue repetitions and more cues attended to. It is possible that these behaviors created a good, less stressful atmosphere for the dogs; therefore, they possibly learnt in a more relaxed manner. An alternative interpretation for these associations was that the trainers behaved more positively (i.e., using neutral or gentle tones of voice) in sessions when the dogs performed better. However, considering the fact that we also recorded reproachful speech used in tandem with good dog performance, and considering studies which have reported the effects of human behavior on their responses (e.g., [26,27,28,30,31,32]), we think this interpretation is improbable. Different emotional responses of dogs to interactions with humans have been demonstrated to be dependent on human behavior/attitude. For example, Horváth and colleagues studied the effects of a 3-min playing session on military dogs with their handlers [63]. Although the researchers instructed all handlers to play with the dogs, some handlers mostly disciplined their animals during the sessions. These animals showed increases in cortisol levels, suggesting a stress response—the opposite outcome to that observed in dogs with handlers who genuinely played with them.

Our results point to a possible effect that vocal trainer behavior may have on animals during training. Some studies have investigated responses of dogs to some types of speech (e.g., [30,32,64]). Gentle speech, and maybe also laughter, fit within a specific type of speech used with dogs, characterized by a high-pitched voice (high frequency) and affectionate content, known as “dog-directed speech”. Dogs prefer when humans use this type of speech to talk to them, demonstrating this by getting closer and looking at them for longer, and both the patterns of rhythm and sound (prosody) and content of the speech matter [31,65]. In our study, when using gentle speech, the trainers praised the dogs, and praise may also function as a type of positive reinforcement [23]. Here, for the first time—as far as we know—we report an association of this type of speech with dog performance during training.

The duration of the trainers’ neutral speech was surprisingly negatively correlated with the duration of the dogs’ orientation to them. One could interpret this result as an aversive response from dogs to neutral speech. This interpretation is possible, but perhaps not likely, given that the same type of speech was associated with a greater duration of tail wagging. Therefore, an alternative—and possibly more sensible—interpretation would be that this result reflects a response not from dogs, but from trainers, who may have used neutral speech for longer when dogs were not directing their attention to them [32,66]. The same rationale can be used to understand the negative correlation between the duration of trainers’ orientation to dogs and the number of cues responded to, and the positive correlation between the time trainers spent oriented to dogs and their latency to respond to them.

We found that the orientation of trainers to dogs was correlated with the dogs’ orientation to them. These results suggest the more focused trainers were on animals, the greater the animals’ attention to them. An interpretation in the opposite direction is also possible (i.e., dog orientation affecting trainer orientation), but the first explanation is in line with studies that found that the trainer’s involvement with the dog during training has an influence on the animal’s attention [51,67]. Dogs seem to perceive the human state of attention, responding faster/more often to cues given by people looking at them [67], and ignoring when the instructor requests a response while looking at someone else instead of the dog. Dogs also prefer to ask for food from people who make eye contact with them than from people who look away [67], which shows that they evaluate the visual attention they receive, being sensitive to human orientation.

Differences in the use of types of speech, as well as the use of laughter, were detected between trainers. Although the number of sessions each trainer conducted differed, this difference in trainer behavior may have contributed to the greater demand for cue repetitions on the part of dogs in sessions with Trainer 2. One possible explanation for the greater use of reproachful instead of gentle speech by Trainer 2 is a lower level of patience. The level of patience, in tandem with the amount of rewards provided, and trainer involvement in the interactions, has been shown to influence animal learning [26]. Trainer 1, who used gentle speech and laughter for longer, obtained responses from the dogs with longer latencies, but these did not prevent dogs from responding to more cues per session than when trained by Trainer 2. Considering the potential effects of a pleasant atmosphere for promoting a positive affective state in dogs [63], our outcomes recommend the use of a soft tone of voice and a relaxed atmosphere in training sessions, with the potential also for improving dogs’ performance.

Our results, although original and relevant, must be interpreted carefully due to some study limitations. Dog breed and age were not balanced in our sample; therefore, we could not investigate the possible effects of these variables on our animals’ responses. Our sample size was limited by the number of shelters that allowed our study to be run in their facilities and in controlled conditions. Nevertheless, small sample sizes are common in dog cognition/learning studies (a review has shown that 57.91% of studies were composed of up to 25 individuals [68]). Although none of the dogs in this study refused to take part in the training interactions, the absence of the CDs’ owners during the sessions may have had an effect on their learning ability or interest in the sessions [69]. Finally, as our training sessions were naturalistic, we cannot draw definitive conclusions about the direction of the associations found between dog and trainer behavior. Future studies to investigate these associations further should test dogs’ responses in sessions with standardized trainer behaviors.

Our study highlighted the possible effects of dog origin on their responses during interactions with humans. Dogs were selected during the domestication process to interact and communicate with humans [4], but a possible side-effect of this selection is a greater ecological and psychological dependence of these animals on human beings. For example, dogs that live inside the house, as family members, show greater dependence on their owners for solving tasks, compared to dogs that live outside [70]. Shelter dogs traditionally experience a life deprived of human contact, and studies like ours suggest that good-quality interactions with humans may be beneficial for them, improving their quality of life. We suggest that shelters should create training programs for their dogs. Such programs, in addition to having the potential to benefit dog welfare [10,71], could increase their chances of being adopted [72]. An increase in the frequency of interactions with humans has been shown to improve shelter dogs’ chances of being adopted [73] by up to 1.4 times [32]. This effect may be attributed to the greater behavioral repertoire developed by these dogs, which is attractive for potential owners. Training programs for shelter dogs may be developed with shelter employees, or even volunteers, at a low or negligible cost.

## 5. Conclusions

Shelter dogs showed better performance and greater excitement during operant conditioning training, compared to CDs. The shelter dogs may have demonstrated greater interest in interactions with people due to their experience of routine social deprivation. Factors related to trainers’ behavior—such as the level of attention and the tone of voice used—were also associated with animals’ responses, suggesting that friendly interactions with humans may benefit their performance and possibly their welfare. These data have the potential to contribute to increasing the effectiveness of dog training, and can also be used as a starting point for further research on the effects of the type of speech used with dogs in their learning process during training.

## Figures and Tables

**Table 1:** Age, sex, weight, breed and origin of the studied shelter dogs (SDs) and companion dogs (CDs).

Dog	Age (in Years) *	Sex	Weight (in Kilograms) *	Breed	Origin
**CD1**	5	Male	6.6	Shitzu	Received from other people
**CD2**	2	Male	2	Mini Maltês	Bought
**CD3**	1	Female	2	Yorkshire	Bought
**CD4**	3	Male	9	Shitzu	Bought
**CD5**	3	Female	8	Mixed breed	Adopted
**CD6**	3	Male	7	Mixed breed	Received from other people
**CD7**	9	Female	6	Lhasa Apso	Bought
**CD8**	5	Male	3.3	Yorkshire Terrier	Found on the streets
**CD9**	6	Female	11	Mixed breed	Adopted
**CD10**	6	Male	11	Shitzu	Found on the streets
**CD11**	10	Female	9	Shitzu/Bichon Frise	Bought
**CD12**	6	Female	7.5	Yorkshire Terrier	Received from other people
**CD13**	1	Male	4.5	Pug	Bought
**CD14**	2	Female	4	Shitzu	Received from other people
**CD15**	12	Male	10	Poodle	Adopted
**SD1**	4	Male	9	Mixed breed	Has lived with a family before (abandoned)
**SD2**	4	Male	10	Mixed breed	Found on the streets
**SD3**	4	Male	8.5	Mixed breed	Found on the streets
**SD4**	4	Female	15.5	Mixed breed	Found on the streets
**SD5**	2	Female	9	Mixed breed	Found on the streets
**SD6**	4	Male	12	Mixed breed	Found on the streets
**SD7**	4	Female	16	Mixed breed	Has lived with a family before (abandoned)
**SD8**	3	Male	9	Mixed breed	Has lived with a family before (abandoned)
**SD9**	3	Male	9	Mixed breed	Has lived with a family before (abandoned)
**SD10**	3	Female	12	Mixed breed	Has lived with a family before (abandoned)
**SD11**	3	Female	12	Mixed breed	Has lived with a family before (abandoned)
**SD12**	5	Female	13.5	Mixed breed	Was born at the shelter
**SD13**	3	Male	15	Mixed breed	Has lived with a family before (abandoned)
**SD14**	2	Male	12	Mixed breed	Found on the streets

* For most SDs these are estimated values.

**Table 2:** Characterization of the vocal behaviors exhibited by trainers with dogs during training sessions, and most frequent contexts of use.

Type of Speech	Characteristics	Context
Gentle speech	Expressions in a high-frequency tone of voice, with gentle content, e.g., “Good boy!”, “You are so beautiful!”	Reinforcement used especially to praise dogs when they responded correctly to a cue request.
Neutral speech	Neutral expressions pronounced calmly, in a neutral tone of voice.	Used mostly to request a response to the cues, and to attract dogs’ attention when they were distracted.
Reproachful speech	Expressions in a neutral or low-frequency tone of voice, with reproachful content, e.g., “No!”, “Stop!”, “Come”, etc.	Used mostly when dogs showed undesirable behavior, such as NTB or biting the trainer’s hand when receiving the snacks.
Laughter	Trainers’ laughter.	When dogs played to get the reward, licked trainers, or tried to reach the reward with their paws without a cue.

**Table 3:** Fixed effects and response variables used in GLM/GLMM models to evaluate the performance and general behavioral responses of dogs during training sessions.

Model	Fixed Effects	Response Variables
GLM1	Dog origin (SD, CD), sex, trainer (T1 or T2)	- Mean latency ^a^ in the responses to the cues at the first request in the last session; - Mean number of cue repetitions ^b^ in the last session; - Number of cues responded to in the last session; - Number of sessions necessary to reach the learning criterion; - Number of sessions necessary to learn each cue; - Number of dogs that reached the learning criteria.
GLMM2	Dog origin (SD, CD), sex	- Mean duration of the exhibition of tail wagging; - Time the animal spent within 1 m of the trainer; - Duration of non-training behaviors; - Time the animal spent oriented towards the trainer.
GLMM3	Trainer (T1 or T2), time trainer spent petting the dog, time trainer spent laughing, time trainer spent using neutral and reproachful speech	- Mean latency in the responses to the cues at the first request; - Mean number of cue repetitions; - Mean number of cues responded to per session.
GLMM4	Trainer (T1 or T2), time trainer spent petting the dog, time trainer spent laughing, time trainer spent using gentle and reproachful speech	- Mean duration of the exhibition of tail wagging; - Time the animal spent within 1 m of the trainer; - Duration of non-training behaviors; - Time the animal spent oriented towards the trainer.

^a^ All latencies and durations were analyzed in seconds. ^b^ If one cue had to be repeated because the animal did not respond to it on the first request, only the response to the last requested cue was considered—all cues that were not responded to were coded as “not executed”.

**Table 4:** Final reduced models (GLM1) of the effects of dog origin, sex, and trainer identity on latency to respond to the cues, cue repetition, and number of cues responded to (in the last training session of each dog); the number of sessions required to learn both commands; the number of sessions required to learn “sit”; the number of sessions required to learn “paw”; and the number of dogs that reached the learning criterion, as recorded during the training sessions of shelter and companion dogs ^1^.

Parameters	Estimate ± SD	z-Value	*p*-Value
**Latency**			
(intercept)	4.420 ± 0.112	39.457	<0.001
Dog origin ^2^	0.095 ± 0.041	2.321	0.02
Trainer ^3^	−0.301 ± 0.047	−6.383	<0.001
Sex ^4^	0.242 ± 0.042	5.741	<0.001
**Cue repetition**			
(intercept)	−0.196 ± 0.384	−0.512	0.61
Dog origin	0.505 ± 0.132	3.833	0.001
Trainer	0.449 ± 0.128	3.488	<0.001
Sex	0.579 ± 0.136	4.253	<0.001
**Cues Responded To**			
(intercept)	4.141 ± 0.189	21.811	<0.001
Dog origin	−0.203 ± 0.075	−2.716	<0.01
Trainer	−0.283 ± 0.086	−3.279	<0.01
Sex	−0.175 ± 0.074	−2.346	0.02

^1^ Parameters not shown were removed during the model selection process. ^2^ Shelter dog or companion dog: shelter dog was the reference group. ^3^ Trainer 1 or Trainer 2: Trainer 1 was the reference group. ^4^ Female or male: female was the reference group.

**Table 5:** Final reduced model (GLMM2) of effects of dog origin and sex on the time spent wagging the tail, time spent within 1 m of the trainer, non-training behaviors, and time spent oriented to trainer, as recorded during all training sessions with shelter and companion dogs ^1^.

Parameters	Estimate ± SD	z-Value	*p*-Value
**Tail wagging**			
(intercept)	11.508 ± 1.559	7.380	<0.001
Dog origin ^2^	−2.365 ± 0.954	−2.479	0.01

^1^ Parameters not shown were removed during the model selection process. ^2^ Shelter dog or companion dog: shelter dog was the reference group.

**Table 6:** Final reduced models (GLMM3) to investigate correlations between trainer identity and behavior and cue repetition, latency in responding to cues, and cues responded to per session during all training sessions with shelter and companion dogs ^1^.

Parameters	Estimate ± SD	z-Value	*p*-Value
**Latency**			
(intercept)	4.885 ± 0.077	62.779	<0.001
Time laughing	0.022 ± 0.002	8.959	<0.001
Time using reproachful speech	−0.087 ± 0.009	−9.571	<0.001
**Cue repetition**			
(intercept)	3.223 ± 0.095	33.734	<0.001
Time laughing	−0.104 ± 0.021	−4.881	<0.001
Time using reproachful speech	−0.070 ± 0.007	−9.922	<0.001
**Cues responded**			
(intercept)	1.864 ± 0.221	8.438	<0.001
Time laughing	0.094 ± 11.595	11.595	<0.001
Time using reproachful speech	0.119 ± 0.023	5.201	<0.001
Time petting	−0.011 ± 0.004	−2.237	0.03

^1^ Parameters not shown were removed during the model selection process. Explanatory variables included in the full models: trainer identity, time petting the dog, time laughing, time using neutral speech, and time using reproachful speech. The time each trainer spent using gentle speech did not fit in these models.

**Table 7:** Final reduced models (GLMM4) of associations between trainer identity and behavior and time spent tail-wagging; time spent within 1 m of the trainer; non-training behaviors (NTBs), and time spent oriented to the trainer, recorded during all training sessions with shelter and companion dogs ^1^.

Parameters	Estimate ± SD	z-Value	*p*-Value
**Tail wagging**			
(intercept)	3.408 ±0.391	8.708	<0.001
Time using neutral speech	0.011 ± 0.001	7.363	<0.001
Time using reproachful speech	−0.113 ± 0.018	−6.123	<0.001
**Time within 1 m**			
(intercept)	5.577 ± 0.033	166.993	<0.001
Time using reproachful speech	−0.045 ± 0.006	−6.694	<0.001
**NTB**			
(intercept)	5.170 ± 0.027	190.550	<0.001
Time using reproachful speech	0.033 ± 0.006	4.918	<0.001

^1^ Parameters not shown were removed during the model selection process. Explanatory variables included in the full models: trainer identity, time petting the dog, time laughing, time using gentle speech, and time using reproachful speech. The time each trainer spent using neutral speech did not fit in these models.

## Data Availability

Data available on request due to restrictions eg privacy or ethical. The data presented in this study are available on request from the corresponding author. The data are not publicly available due to privacy restrictions—although dog owners have signed a free and informed consent for the study of their dogs, we prefer to make videos available on request.

## Supplementary Materials

The following are available online at https://www.mdpi.com/article/10.3390/ani11051360/s1, Table S1: Generalized Linear Model of the effect of dog size (weight) on dog performance (Number of sessions to learn cues, Number of dogs that reached learning criterion, Cues answered, Cue repetition, Latency to respond to the cues), recorded during the last training session of shelter and companion dogs.
