# Supplementary material for: Can Dogs’ Origins and Interactions with Humans Affect Their Accomplishments? A Study on the Responses of Shelter and Companion Dogs during Vocal Cue Training
Source: Animals (Basel). 2021 May 11;11(5):1360. doi: 10.3390/ani11051360 (PMC8151446; doi:10.3390/ani11051360)
Supplement: Supplementary file 1 [file animals-11-01360-s001.zip › animals-1132424-supplementary.pdf]

Table S1. Generalized Linear Model of the effect of dog size (weight) on dog performance (Number of sessions to learn cues, Number of dogs that reached learning criterion, Cues answered, Cue repetition, Latency to respond to the cues), recorded during the last training session of shelter and companion dogs.

**Parameters**	**Estimate ± SD**	**z-value**	***p***
(intercept)	1.69905 ± 0.21929	7.748	<0.0001
Sessions	-0.01149 ± 0.02271	-0.506	0.61
(intercept)	0.66145 ± 0.36760	1.799	0.07
Dogs that learnt	-0.01076 ± 0.03803	-0.283	0.78
(intercept)	3.33430 ± 0.09761	34.160	<0.0001
Cues answered	-0.01423 ± 0.01014	-1.403	0.16
(intercept)	2.068630 ± 0.171283	12.077	0.61
Cue repetition	0.008917 ± 0.017291	0.516	0.6
(intercept)	4.525235 ± 0.056192	80.532	0.61
Latency	0.004433 ± 0.005916	0.749	0.45
